# *Leclercia adecarboxylata* Cholecystitis with Septic Shock in Immunocompetent Patient

**DOI:** 10.1155/2019/5057071

**Published:** 2019-08-04

**Authors:** Nooraldin Merza, John Lung, Ahmed Taha, Ahmed Qasim, Jill Frost, Tarek Naguib

**Affiliations:** ^1^Department of Internal Medicine, Texas Tech University Health Sciences Center, Amarillo, TX, USA; ^2^School of Medicine, Texas Tech University Health Sciences Center, Amarillo, TX, USA; ^3^School of Pharmacy, Texas Tech University Health Science Center, Amarillo, TX, USA

## Abstract

*L. adecarboxylata *is a Gram-negative rod previously named* Escherichia adecarboxylata*, isolated as normal flora in the gut of animals including human stool. Most reported cases refer to immunocompromised patients with polymicrobial infections and water environments. Here we present a case of 51-year-old immunocompetent female presented with nausea, vomiting, malaise, and subjective fever for few days. On examination, she was drowsy but arousable and oriented to person, place, time, and situation. Her abdomen was tender globally and more tender in the epigastric area. Vitals showed a temperature of 37°C, pulse of 110 beats/min, blood pressure of 75/50 mmHg, and oxygen saturation of 91% on room air. An HIV panel and hepatitis panel were negative. Liver and gallbladder ultrasound was performed, revealing multiple nonmobile stones with shadowing noted within the gallbladder sac, a thickened gallbladder wall, and a moderate amount of pericholecystic fluid. Broad spectrum antibiotics, crystalloid fluids, and vasopressors were initiated. A few hours after admission she developed respiratory failure for which she underwent endotracheal intubation. An ultrasound guided gallbladder drain was performed. Culture of the biliary fluid yielded pure growth of pan-sensitive* L. adecarboxylata*; antibiotics were narrowed accordingly. The patient was on the maximum doses of vasopressin, norepinephrine, and epinephrine with a blood pressure of 75/45 and a mean arterial pressure of 51. She passed away on the fourth day of admission.

## 1. Background

*Leclercia adecarboxylata* is a unique Enterobacteriaceae member frequently isolated from water with infrequent case reports in the literature. Originally described by Leclerc in 1962 as* Escherichia adecarboxylata*, these facultative aerobic, Gram-negative bacilli were reclassified by the development of more accurate identification methods as a member of the Enterobacteriaceae family [1]. This bacterium is usually associated with polymicrobial infection in immunocompromised individuals, although there are recent observations of finding this microorganism in healthy individual. We hereby report a unique case of cholecystitis leading to septic shock caused by* Leclercia adecarboxylata* in an immunocompetent individual without significant water exposure.

## 2. Case Report

**Figure 1:**
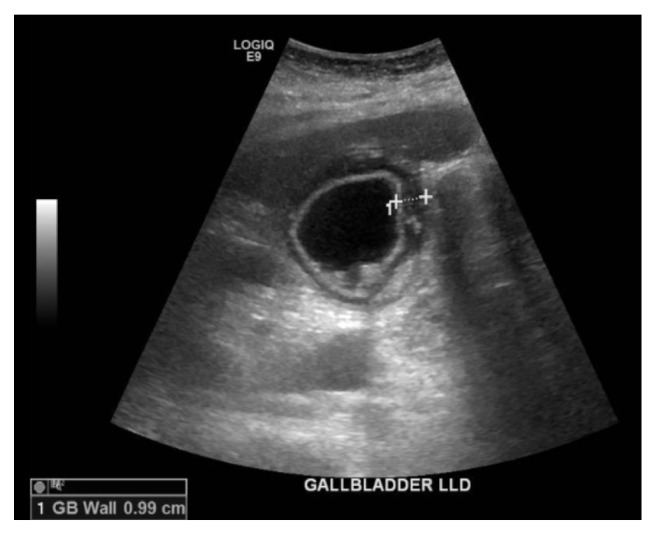
Ultrasound of gallbladder showing inflamed gallbladder with multiple nonmobile stones with shadowing in the gallbladder body. The gallbladder wall is thickened to 0.99 cm. A moderate amount of pericholecystic fluid is identified.

A 51-year-old female presented to the emergency department with nausea, vomiting, malaise, and subjective fever for few days. She reported difficulty with ambulation as well as right hip and back pain for several months, with nonsignificant past medical or surgical history. She denied alcohol, drug, or tobacco use. She also denied any recent travel, sick contacts, or exposure to standing water. On physical examination, she was drowsy but arousable and oriented to person, place, time, and situation. Her abdomen was tender globally and more tender in the epigastric area. Vitals showed a temperature of 37°C, pulse of 110 beats/min, blood pressure of 75/50 mmHg, and oxygen saturation of 91% on room air. Labs are shown in Table 1. An HIV panel, hepatitis panel, ceruloplasmin, antinuclear (ANA) panel, smooth muscle antibody, anti-centromere antibody, antichromatin antibody, anti-DNA antibody, anti-Jo-1 antibody, anti-Scl-70 antibody, anti-SSA antibody, and anti-SSB antibody were all negative. Alpha-1 antitrypsin was slightly above normal limits at 204 mg/dl (reference 90-200). Imaging revealed a normal chest X-ray. Electrocardiogram showed sinus tachycardia with no ischemic changes. A CT scan of the abdomen showed a gallbladder wall thickening and multiple gallstones. To exclude cholecystitis, a liver and gallbladder ultrasound. (Figure 1) was performed, revealing multiple nonmobile stones with shadowing noted within the gallbladder sac, a thickened gallbladder wall, and a moderate amount of pericholecystic fluid.

**Figure 2:**
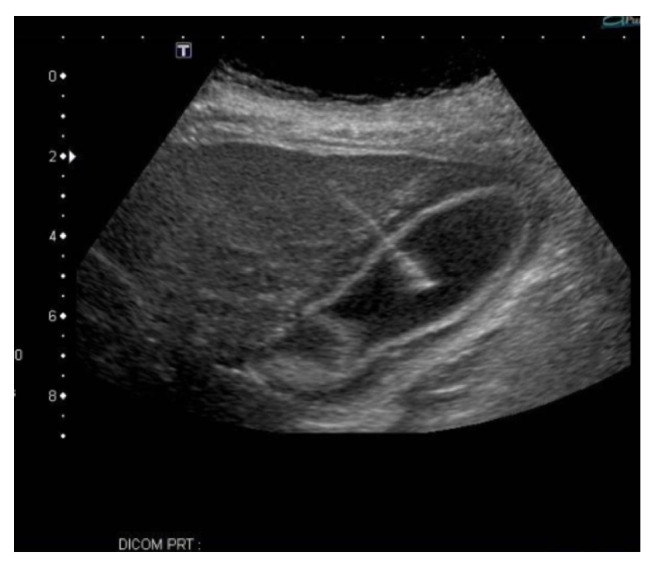
IR-guided cholecystostomy tube placement and drainage and irrigation of the gallbladder with 60 cc of black bile aspiration.

Broad spectrum antibiotics (IV vancomycin and piperacillin/tazobactam), crystalloid fluids, and vasopressors were initiated. A few hours after admission she developed respiratory failure for which she underwent endotracheal intubation. Due to her shock state she was not a candidate for surgery. An ultrasound guided gallbladder drain (Figure 2) was performed. Culture of the biliary fluid yielded pure heavy growth of pan-sensitive* L. adecarboxylata*; antibiotics were narrowed accordingly to IV meropenem.

**Figure 3:**
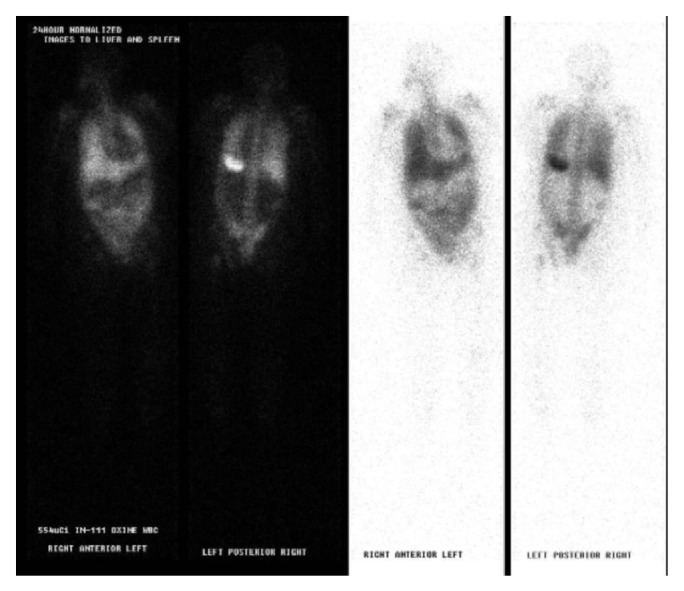
Indium-111 white blood cell scan showing no localization of abscess and atypical diffuse pulmonary uptake of white blood cells, which can be associated with an opportunistic infection, pulmonary drug toxicity, and sepsis.

On the second day of admission to the intensive care unit the patient developed signs of disseminated intravascular coagulopathy from septic shock and acute liver failure with profound lactic acidosis and deteriorated rapidly. The patient developed skin mottling of all four extremities due to limb ischemia likely due to peripheral artery vasoconstriction secondary to shock and vasopressor use. The patient had no Doppler signals in the bilateral radial, ulnar, dorsalis pedis, and posterior tibial arteries. Indium-111 tagged WBC scan was performed (Figure 3) to make sure we have not missed another source of infection. This showed diffuse pulmonary uptake which we felt was due to acute respiratory distress syndrome and there was no abscess. The patient received disseminated intravascular coagulation (DIC) treatment including multiple packed red blood cells (PRBC), cryoprecipitate and fresh frozen plasma (FFP) transfusions, and treatment for septic shock including antibiotics and vasopressors.

The patient also received hemodialysis due to acute kidney injury secondary to septic shock. Consults with gastroenterology and general surgery resulted in recommendations of no surgical intervention because of the critical condition of the patient with intubation, mechanical ventilation, hemodialysis, and vasopressors. The patient developed pulseless electrical activity (PEA) cardiac arrest but recovered after one round of cardiopulmonary resuscitation and 1 dose of epinephrine. The patient was on the maximum doses of vasopressin, norepinephrine, and epinephrine with a blood pressure of 75/45 and a mean arterial pressure of 51.

The patient developed another PEA cardiac arrest 11 hours later and could not be resuscitated despite aggressive resuscitation measures that followed advanced cardiac life support protocol. She passed away on the fourth day of admission.

## 3. Discussion

*L. adecarboxylata *is a motile, Gram-negative rod previously named* Escherichia adecarboxylata*, isolated as normal flora in the gut of animals including human stool. Most reported cases refer to immunocompromised patients which gives a clear suggestion of the exclusivity of* L. adecarboxylata *as an opportunistic pathogen associated with polymicrobial infections and water environments, particularly as a contaminant in polluted water [2, 3]. The paucity of published case reports of human infections with this bacteria may reflect misdiagnosis, as the organism shares many biochemical features with* E. coli*, rather than a true infrequency of human infection [4].* L. adecarboxylata *causes infections at various organs including pharyngeal abscess, pneumonia, cholecystitis, gluteal abscess, and cellulitis.

There are at least 74 cases reported in English-language journals since the first case report of* L. adecarboxylata *in 1991, including 19 cases in immunocompetent patients and two cases in patients with cholecystitis, one chronic and one acute (Tables 2 and 3) [2, 4–22].

There is one case in the literature of an 81-year-old female (Table 3) with a past medical history of metabolic syndrome and chronic atrial fibrillation who presented with acute cholecystitis and had a positive* L. adecarboxylata *biliary fluid culture [10]. The patient in that case report had risk factors for a compromised immune system including old age, cardiovascular risk factors, and metabolic syndrome, while our patient was immunocompetent and did not have any known source for contracting* L. adecarboxylata*. Our patient tested negative for HIV and hepatitis B and C virus. The team ordered a tagged WBC scan to make sure we are not missing another hidden infection.

*L. adecarboxylata *is not commonly associated with sepsis and rapid deterioration requiring mechanical ventilation and resuscitative measures as in this case scenario. There is one case report of a patient who developed* L. adecarboxylata *sepsis, required intubation, and then died in the hospital. This patient had a viral infection prior to admission and then developed pneumonia due to a multidrug-resistant* L. adecarboxylata *infection [23]. Our patient who developed septic shock had a* L. adecarboxylata *infection was pan-sensitive to most of the tested antibiotics, with no immunocompromising risk factors. Most* L. adecarboxylata *infections are sensitive to many antibiotic options and frequently respond to treatment without needing to switch antibiotics [24].

*Most of L. adecarboxylata *strains are naturally sensitive to tetracyclines, aminoglycosides, all but two *β* lactams, quinolones, folate pathway inhibitors, chloramphenicol, nitrofurantoin, and azithromycin. They are naturally resistant to penicillin G, oxacillin, erythromycin, roxithromycin, clarithromycin, ketolides, lincosamides, streptogramins, linezolid, glycopeptides, rifampicin, fusidic acid, and fosfomycin, with only minor medium-dependent differences in susceptibility to most antibiotics [24, 25].

However, multiantibiotic resistant* L. adecarboxylata *has been reported in the literature [21, 26], illustrating the need to continue to practice good antibiotic stewardship to prevent development of resistant strains.

The uniqueness of this case could be summarized by the presentation of septic shock with* L. adecarboxylata *in an immunocompetent state. There are only a few reported cases of* L. adecarboxylata *infection in immunocompetent patients [5]. Because* L. adecarboxylata *is typically found in polymicrobial infections, it is rare to encounter the growth of pure cultures as in this case. There are few reported cases of pure cultures in immunocompetent patients, but not with this severity [5, 22].* L. adecarboxylata *cases in immunocompetent patients are associated with exposed wound infections and contact with water environments [2], while our patient did not have any open wound and lived in a dry, flat area with no open water sources.

## 4. Conclusion

*L. adecarboxylata *is a rare Gram-negative rod which often infects immunocompromised hosts. We report a case of acute cholecystitis caused by* L. adecarboxylata *leading to profound septic shock with DIC and liver failure that did not respond to aggressive treatment measures. Our case illustrates an opposite picture of what has long been known as an opportunistic organism and a cause of mild infections. We hope by presenting this case we contribute to the current knowledge about this organism and perhaps increase the reporting and identification of the organism as a potential invasive pathogen.

## Figures and Tables

**Table 1:** The patient's lab on days 1, 2, 3, and 4.

	Day 1	Day 2	Day 3	Day 4
WBC (x10e3/mcL)	9.1	7.6	12.1	13.8

Neutrophils (x10^3^/mcL)	6.6	5.6	10.1	12.4

Hemoglobin (gm/dL)	11.4	8.2	8.2	7.7

Platelets (x10e3/mcL)	136	114	45	54

INR	1.52	2.2	2.7	3.0

Fibrinogen (mg/dL)		172	61	75

D-Dimer (mg/L)	>35	>35	>35	>35

LDH (units/L)	>4000	7898	9738	

Troponin (ng/mL)	0.088			

Glucose (mg/dL)	45	71	117	220

Na (mmol/L)	134	144	145	139

K (mmol/L)	6.9	6.4	4.5	3.9

BUN (mg/dL)	73	63	40	14

Creatinine (mg/dL)	2.4	2.4	2.1	1.2

CO2 (mmol/L)	8	4	11	22

Anion Gap (mmol/L)	22	29	30	13

Lactate (mmol/L)	11.5	17.5	20	10

AST (units/L)	1650	10623	17523	5846

ALT (units/L)	553	1953	2561	1823

ALP (units/L)	260	257	333	409

Bilirubin	0.98	2.1	2.6	4.8

Albumin	2.5	3	2.5	2.2

**Table 2:** Clinical case reports of *L. adecarboxylata* infections in immunocompetent patients.

Case	Age	Gender	Infection site and clinical information	Co-infection	Outcome	Identification method
Temesgen et al. 1997	35	M	right foot would culture, s/p crush injury	*Enterobacter cloacae, Citrobacter freundii, Enterococcus species, Klebsiella pneumoniae, Stenotrophomonas maltophilia, and Corynebacterium, Acremonium, Penicillium, Mucor, Geotrichum*	Recovery	Biochemical testing

Temesgen et al. 1997	23	M	left toe wound culture, s/p nail puncture	*Acinetobacter calcoaceticus, Enterobacter agglomerans*	Recovery	Biochemical testing

Greco et al. 2001	75	M	wound culture in right hand	*Staphylococcus epidermidis*	Recovery	Not mentioned

Pérez-Moreno et al. 2003	60	M	synovial fluid, infection arthritis	None	Recovery	Biochemical testing

Hess et al. 2008	40	F	right foot wound culture, s/p superficial incision and swimming in pool	None	Recovery	Biochemical testing

Dalamaga et al. 2009	53	M	left arm wound culture, s/p chemical injury	None	Recovery	Biochemical testing

Mbamalu and Macariola 2011	11 mo	M	blood bacteremia	None		Not mentioned

Bali et al. 2013	32	M	peritonsillar and lateral pharyngeal abscess	None	Recovery	Not mentioned

Zapor et al. 2013	25	M	pus and necrotic tissue in right buttock, s/p wartime IED explosion	None	Recovery	16s ribosomal sequencing with 16s rRNA gene

Haji et al. 2014	70	M	blood culture, infectious arthritis	None	Recovery	Not mentioned

Keren et al. 2014	46	M	left foot soft tissue infection, s/p laceration while surfing	*Enterobacter cloacae*	Recovery after 2 weeks	VITEK-2 automated microbial- identification system

Anuradha 2014	50	M	gluteal abscess	None	Recovery	MicroScan autoSCAN-4 automated microbial identification system

Anuradha 2014	31	F	vaginal swab, vaginitis	None	Recovery after 10 days	MicroScan autoSCAN-4 automated microbial identification system

Grantham et al. 2015	9	F	left lower extremity soft tissue infection s/p penetrating injury	None	Recovery after 2 months	Not mentioned

Hurley et al. 2015	2	M	right thumb soft tissue infection s/p laceration	None	Recovery	VITEK automated microbial identification system

Prakash et al. 2015	32	F	tracheal aspirate, hospital-acquired pneumonia	None	Recovery after 1 week	VITEK automated microbial identification system

Allawh, Camp 2015	26	F	fingernail soft tissue infection s/p trauma to nail	*Staphylococcus*	Not symptomatic after 2 weeks	Not mentioned

Riazzo et al. 2017	30	M	Subcutaneous trauma injury to foot	*Klebsiella pneumoniae, Pseudomonas*	Discharge after 3 months	Microscan Walkaway, confirmed by MALDI-TOF

Capretta et al. 2018	9	M	right leg soft issue infection, cellulitis s/p fall	N/A	Recovery after 3 weeks	Not mentioned

**Table 3:** Clinical case reports of *L. adecarboxylata *infections in patients with cholecystitis who were also immunocompromised.

Case	Age	Gender	Infection site and clinical information	Co-infection	Outcome
de Baere et al. 2001	78	F	Chronic cholecystitis	*Enterococcus*	Recovery

Jover-Sáenz et al. 2008	81	F	bile, cholecystitis	None	Recovery after 1 month
